# Postural control deficits due to bilateral pyramidal tract lesions exemplified by hereditary spastic paraplegia (HSP) originate from increased feedback time delay and reduced long-term error corrections

**DOI:** 10.3389/fnhum.2023.1229055

**Published:** 2023-12-05

**Authors:** Daniela Dalin, Isabella Katharina Wiesmeier, Bernhard Heimbach, Cornelius Weiller, Christoph Maurer

**Affiliations:** ^1^Department of Neurology and Neurophysiology, Medical Faculty, University Medical Center, University of Freiburg, Freiburg im Breisgau, Germany; ^2^Department of Psychiatry and Psychotherapy, University of Tübingen, Tübingen, Germany

**Keywords:** postural control, pyramidal tract, sensorimotor system, hereditary spastic paraplegia, model, exercise

## Abstract

Pyramidal tract lesions determine the clinical syndrome of Hereditary Spastic Paraplegia (HSP). The clinical impairments of HSP are typically exemplified by their deficits in mobility, leading to falls and injuries. The first aim of this study was to identify the cause for postural abnormalities caused by pyramidal tract lesions in HSP. The second aim was to specify the effect of treadmill training for postural abnormalities. We examined nine HSP patients before and after treadmill training, as well as nine healthy control subjects during perturbed and unperturbed stance. We found that HSP was associated with larger sway amplitudes and velocities. Body excursions following platform tilts were larger, and upper body excursions showed a phase lead. Model-based analysis detected a greater time delay and a reduced long-term error correction of postural reactions in the center of mass. HSP patients performed significantly better in clinical assessments after treadmill training. In addition, treadmill training reduced sway amplitudes and body excursions, most likely by increasing positional and velocity error correction gain as a compensatory mechanism, while the time delay and long-term error correction gain remained largely unaffected. Moreover, the upper body’s phase lead was reduced. We conclude that HSP leads to very specific postural impairments. While postural control generally benefits from treadmill training, the effect seems to mainly rely on compensatory mechanisms, whereas the original deficits are not affected significantly.

## 1 Introduction

Hereditary Spastic Paraplegia (HSP) impairs full body posture leading often to falls and injuries (Nonnekes et al., 2017). The clinical presentation of HSP is mainly attributable to pyramidal tract lesions. Among different forms of the disease, mere lower limb spasticity and bladder disturbances are described as “pure” or “uncomplicated,” whereas they are classified as “complicated” when associated with other neurological signs (Salinas et al., 2008; Fink, 2013). Age of symptom onset, rate of progression, and degree of disability vary among the types of HSP, as well as within individual families with precisely the same gene mutation (Fink and Hedera, 1999; Fink, 2013).

The clinical assessment of pyramidal tract lesions is usually based on exaggerated tendon reflexes and muscle hypertonia in relaxed patients (Dietz and Sinkjaer, 2007). There is evidence, however, that these assessments correlate weakly with functional deficits of stance and gait (e.g., Burne et al., 2005; Dietz and Sinkjaer, 2007; in stroke patients, Nardone et al., 2001).

Neurophysiological correlates of pyramidal tract lesions have been described as impaired control of supraspinal drive and the use of afferent input, impaired reflex activity modulation, and abnormally prolonged motor conduction time (Jørgensen et al., 2005; Dietz and Sinkjaer, 2007). Secondary anomalies in muscle fibers might compensate for paresis and allow functional movements on a simpler organizational level (Dietz and Sinkjaer, 2007). However, the functional correlates of pyramidal tract lesions-induced postural impairments remain unknown.

Attempts to characterize pyramidal tract lesions from a more technical perspective include measuring joint torque increases or stretch reflex responses (Le Cavorzin et al., 2001; Alibiglou et al., 2008; Calota and Levin, 2009). Recent studies addressed the objective evaluation of gait in HSP patients (e.g., Bonnefoy-Mazure et al., 2013; Serrao et al., 2016). Again, the impact on postural impairments remains unclear.

Research on exercise therapies revealed that treadmill training improves gait parameters in patients with pyramidal tract lesions caused by various neurological diseases, i.e., cerebral palsy (Hodapp et al., 2009), stroke (Mehrholz et al., 2017) or spinal cord injury (Phadke et al., 2007). Pohl et al. (2002) advanced treadmill training by establishing a “Structured Speed-Dependent Treadmill Training” (STT). It proved to be superior to classic treadmill or conventional gait training to improve gait parameters, whereas effects on postural stability are still unknown.

Clinical assessments of postural control include the examination of stance and gait, usually in combination with absent or altered sensorimotor information (e.g., Romberg’s Test), but clinicians still have no standard means of measuring postural control.

Few studies have evaluated HSP patients’ postural control (Nardone et al., 2001; Nonnekes et al., 2013), and most evaluated pyramidal tract lesions in diseases affecting one limb or the limbs on one side, e.g., stroke-induced. We can assume that pyramidal tract lesions affecting both legs may lead to diverse study results as patients cannot rely on one healthy limb.

There is evidence that a perturbation-based approach enables the identification of sensorimotor parameters like response gain and phase, upper vs. lower body strategy, sensorimotor latencies, and sensory channels involved in mechanisms of postural control. It is based on the relationship between external perturbations of the body support surface and the human body’s reaction in space (Engelhart et al., 2014; Pasma et al., 2014; Wiesmeier et al., 2017).

In this pilot study we aimed to identify underlying mechanisms of postural deficits in “uncomplicated” HSP patients as a typical example for an impaired pyramidal tract taking a perturbation-based approach. We expected that HSP patients would differ in their postural-control mechanisms from healthy subjects. Moreover, we hypothesized that postural abnormalities would be well characterized by model-based analysis of externally perturbed stance. Finally, we hypothesized that HSP patients’ postural control is modulated by STT and that beneficial effects of treadmill training might not be confined to gait parameters, but also encompass postural stability.

## 2 Patients and methods

### 2.1 Experimental design and statistical analysis

Nine HSP patients (6 male, 3 female) with a mean age of 55.2 ± 5.3 years (± SD) participated in this pilot study. They suffered from “uncomplicated” HSP with late onset (mean age of onset 43.2 ± 8.6 years; ± SD) and a mean disease duration of 12 ± 6.4 years (± SD). Six patients had a known genetic defect leading to HSP (see Table 1). The other 3 patients were considered to suffer from a sporadic form or yet unknown mutation. All patients were able to walk with different walking aids. Table 1 summarizes their clinical information.

**TABLE 1:** Clinical characteristics of the HSP group.

Patient No.	Age (years)	Genetic phenotype	Age at disease onset	Disease duration (years)	Years since diagnosis
1	53	−*	33	20	13
2	66	SPG 4	54	12	3
3	59	SPG 4	48	11	11
4	50	−*	46	4	4
5	52	SPG 4	30	22	13
6	56	SPG 4	40	16	5
7	52	−*	46	6	3
8	59	SPG 10	54	5	4
9	50	SPG 4	38	12	10
**Mean**	**55.2**	**5x SPG 4**	**43.2**	**12.0**	**7.3**
**SD**	**5.3**		**8.6**	**6.4**	**4.3**

SD, standard deviation. *No known genetic phenotype found.

The control group consisted of nine healthy subjects who did not differ significantly from the HSP patients in terms of age, body height, body mass and body mass index (BMI, *p* > 0.05).

All patients and subjects gave their written informed consent in accordance with the Declaration of Helsinki. The study protocol was approved by the Ethics Committee of the University of Freiburg (IRB # 256/01).

### 2.2 Clinical assessments

Patients were examined thoroughly by an experienced neurologist (BH), including tests with the Rydal-Seiffer tuning fork. We also assessed nerve conduction time and somatosensory evoked potentials in all but two patients who were within the normal range. Patients also completed standardized questionnaires and the testing of motor skills (see below) before and after treadmill training. None of the patients suffered from ataxia, peripheral neuropathy, or parkinsonism.

Our exclusion criteria comprised the features of complicated HSP: the degenerative process affects multiple parts of the nervous system and presents clinically with additional features like neuropathy, ataxia, cognitive impairment, seizures, optic atrophy, amyotrophy, and extrapyramidal involvement. We scored the following features in particular: mental retardation, dementia, psychosis, epilepsy, visual loss, cataract, gaze evoked nystagmus, dysarthria, dysphagia, limb ataxia, gait ataxia, extrapyramidal motor signs, muscle wasting UEX, muscle wasting LEX, loss of reflexes UEX (upper extremity), loss of reflexes LEX (lower extremity), impaired touch sense, impaired pinprick sensation, impaired vibration sense, impaired joint position sense, impaired temperature discrimination, facial dysmorphism, skin abnormalities, skeletal abnormalities, Botox in the last 3 months. We had to rule out one patient with additional peripheral neuropathy.

The severity of spastic paraplegia was quantified via the Spastic Paraplegia Rating Scale (SPRS, Schüle et al., 2006). This scale rates spasticity, muscle weakness, contractures and pain as well as typical activities of daily living prone to being affected by HSP. We also employed the Barthel index to assess restrictions of daily living activities (Mahoney and Barthel, 1965).

According to the modified Ashworth scale (Bohannon and Smith, 1987; Pandyan et al., 1999), spasticity was rated for hip, knee, upper ankle joint and toes and averaged for each subject.

Motor function was further assessed with the Tinetti Balance and Gait Test (Tinetti, 1986) which is commonly used in clinical practice and addresses gait and balance in two subscales.

Patients also performed the Timed Up-and-Go Test (TUG, Podsiadlo and Richardson, 1991) used to examine the time needed to rise from a chair, walk three meters straight, turn around, walk back and sit down. We also measured the maximum walking speed and maximum distance on the treadmill during 30 min walking at the second and the last training session.

### 2.3 Procedures

Details of the experimental procedures are similar to Wiesmeier et al. (2015).

**FIGURE 1:**
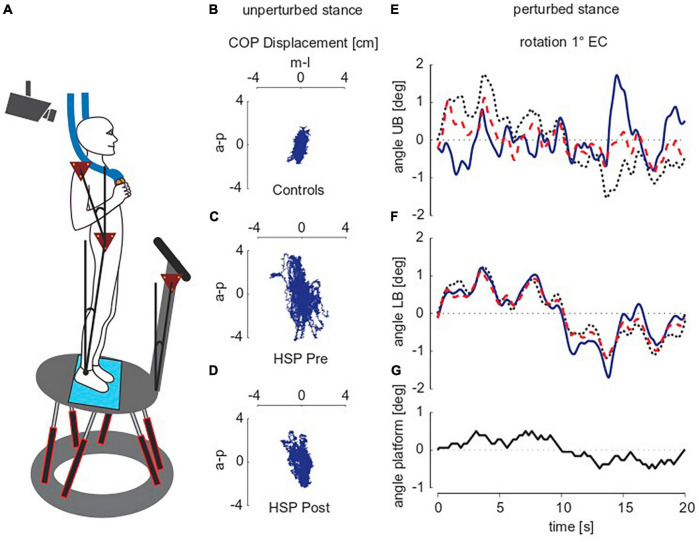
Experimental setup and raw traces of unperturbed and perturbed stance. **(A)** Subject standing on the platform. **(B–D)** Exemplarily illustrate the raw traces of 2D center of pressure (COP)-movements during unperturbed stance of two representative subjects, one of the control group **(B)** and one of the HSP group before [pre; **(C)**] and after [post; **(D)**] treadmill training while standing with closed eyes. a-p, anterior-posterior, m-l, medio-lateral, HSP, Hereditary Spastic Paraplegia. **(E–G)** Show the pseudo random stimulus **(G)** and group averages for upper body [UB; **(E)**] and lower body [LB; **(F)**] angular excursions in degrees (deg) during perturbed stance. Shown are representative subjects of each the control group (black dotted line), and the HSP group before (blue line) and after training (red dotted line). The stimulus amplitude of platform rotations was one degree, subjects stood with eyes closed (EC). Note that the average time series differ mainly at maximum body excursions.

Subjects stood (as naturally and comfortably as possible) upright on a custom-built motion platform, stance width was adapted to shoulder width (Figure 1A). For security reasons, all subjects held two handles in their hands that were attached to ropes hanging loosely from the ceiling. The handles and ropes enabled no spatial orientation (Buettner et al., 2017). Unperturbed stance was investigated on the stationary platform. Subjects performed six trials distributed into three repetitions, each with eyes open and with eyes closed. One trial consisted of 2 minutes of quiet standing with pauses for rest in between.

Perturbed stance was assessed by the subjects’ postural responses to transient tilts of the moving platform in the anterior-posterior direction (a-p, sagittal plane). Figure 1G shows the time course of the tilt stimulus, which relies on a pseudorandom ternary sequence of numbers (PRTS). One stimulus cycle lasted 20 s and was repeated six times (during each 2 min-trial). Peak-to-peak amplitude was either 0.5 or 1°. During the six trials (three repetitions per amplitude), subjects kept their eyes closed. The HSP group performed the platform experiments before and after 10 days of intensive treadmill training, whereas the control group underwent the platform experiments only once.

### 2.4 Data acquisition and analysis

During unperturbed stance, we recorded the two-dimensional center of pressure (COP) sway path with the help of a force transducing system (Kistler platform type 9286, Winterthur, Switzerland^®^). Figures 1B–D shows the COP sway path of one representative control subject and the same HSP patient before and after training. Root mean square (RMS; sway amplitude), Mean Velocity (MV; sway velocity) and 50%-frequency of body sway (F50; frequency content of sway) were extracted from COP measures using custom-made software programmed in MATLAB^®^ (The MathWorks Inc., Natick, MA, USA). All values were calculated for single subjects and subsequently summarized in a group mean.

Furthermore, we measured the position of the body segments in space using an optoelectronic device with markers placed at the subjects’ shoulder and hip and on the platform (Optotrak^®^ 3020, Waterloo, ON, Canada). Optotrak and Kistler outputs as well as stimulus signals were recorded with software programmed in LabView^®^ (National Instruments, Austin, TX, USA).

We obtained upper body (UB) and lower body (LB) excursions in space from 3-D translational and angular positions of each marker. Stimulus-response data on perturbed stance was calculated using a discrete Fourier transformation (DFT) in MATLAB. From Fourier coefficients of stimulus and response time series, we calculated transfer functions, i.e., gain, phase and coherence values across five cycles in the stimulus presentation. The first cycle was discarded due to transient posture adjustments before reaching a steady state (Figures 1E, F). Gain values relate the body’s response (postural reaction; upper and lower body angle) to the stimulus (external perturbation; platform angle). For example, a gain value more than one indicates amplified external perturbation. Phase is the relative delay between the stimulus and body’s reaction. For example, phase advance is indicated by more positive phase values. Coherence helps to estimate the linear relationship between stimulus and response. A coherence value of zero implies no linear relationship, whereas a coherence value of one implies a perfect linear relationship. Gain, phase and coherence depend on frequency (see Peterka, 2002).

### 2.5 Parameter identification technique

Transfer functions were implemented into an established biomechanical model of human stance (e.g., Maurer and Peterka, 2005; Engelhart et al., 2014). The human body is modeled as an inverted pendulum with the center of rotation at the ankle joint. Deviations from an upright position are fed back into the model via vestibular and proprioceptive sensors. A weighting mechanism determines the individual importance of the information from the proprioceptive system (Wp). A time delay (Td) takes into account the human sensory-motor-conduction and central processing time. The inverted pendulum is stabilized via a PID controller mimicking active stiffness (Kp), damping (Kd), and integral properties (Ki) of the body. Moreover, passive stiffness (Ppas) and passive damping (Dpas) are included in the model. Using a MATLAB-based script, the model’s settings are optimized with the “fmincon” function until the mean standard error (mse) between measured and modeled transfer functions is minimal.

### 2.6 Treadmill training

Our study’s HSP group performed a “Structured Speed-Dependent Treadmill Training” (STT) according to Pohl et al. (2002) lasting 30 min every day over a 2-week period. Adapted to each patient’s walking ability, 5 patients received 20% body weight support (BWS) during treadmill training. Four patients with BWS needed intermittent help with foot placement too.

### 2.7 Statistical analyses

We performed statistical analyses with Microsoft Excel and JMP^®^ (SAS Institute Inc., Cary, NC, USA). First, we tested the normal distribution with the Kolmogorov-Smirnov test. Statistical significance between HSP patients before treadmill training and the healthy control group was then examined applying analyses of variance (ANOVA) with the between-subjects variable “group” (HSP patients, control subjects). For unperturbed stance, we chose visual condition (eyes open, eyes closed) and sway direction (mediolateral, anteroposterior) as within-subjects variables. For externally perturbed stance we applied stimulus amplitude (0.5 and 1°) and body segment (hip, shoulder) as within-subjects variables. We also tested the effect of treadmill training on postural control of HSP patients via multivariate analyses of variance (MANOVA) with “time” as the repeated measure variable. Statistical significance was assumed at *p* ≤ 0.05. To estimate the effect of treadmill training on clinical assessments, we adjusted the significance levels for multiple comparisons.

## 3 Results

### 3.1 Clinical assessments

Table 2 shows an overview of our clinical assessment findings. Prior to treadmill training (pre) the average Spastic Paraplegia Rating Scale (SPRS) amounted to 21.1 ± 8.2 (mean ± SD; 52 points = maximal impairment), after treadmill training (post) the SPRS was significantly reduced (17.8 ± 7.1; *F* = 7.6, *p* = 0.03). Treadmill training significantly reduced the average Ashworth scale score of 1.8 ± 0.9 before training to 1.5 ± 0.8 (both “slight increase in muscle tone”; *F* = 10.3, *p* = 0.01). Treadmill training also significantly increased the maximum walking distance (pre 1104 ± 279 m, post 1669 ± 665 m; *F* = 6.3, *p* = 0.04) and maximum walking speed (pre 2.7 ± 0.8 m/s, post 3.8 ± 1.3 m/s, *F* = 15.6, *p* = 0.006), whereas it only slightly reduced the time needed to do the Timed Up and Go Test (TUG, pre 21.7 ± 12.8 s, post 19.4 + /10.7 s; *F* = 0.5, *p* = 0.5). Moreover, it ameliorated slightly the performance of patients in the Tinetti Balance and Gait Test, thereby lowering the risk of falls (total score pre 17.3 ± 5.3, “high risk for falls,” post 19.8 ± 4.5, “risk for falls”; *F* = 4.8, *p* = 0.06; Table 2). Seven of the nine patients scored 100 in the Barthel index, one 95 and one 90. At the end of treadmill training, the number of patients needing body weight support (BWS) was reduced from five to two. All patients were able to walk independently; help with foot placement was no longer necessary.

**TABLE 2:** Clinical assessments.

Pat. no.	SPRS sum	SPRS sum	ASH mean	ASH mean	Tinetti sum	Tinetti sum	Max Vel. (m/s)	Max Vel. (m/s)	Max Dist. (m)	Max Dist. (m)	TUG (s)	TUG (s)
	**Pre**	**Post**	**Pre**	**Post**	**Pre**	**Post**	**Pre**	**Post**	**Pre**	**Post**	**Pre**	**Post**
1	38	28	2.67	1.67	6	15	2.1	3.2	700	1210	55	34.6
2	13	12	1.33	1.00	19	21	2.6	4.2	1280	1460	21.7	17.9
3	25	21	2.67	2.33	16	16	2.7	3.1	1070	1518	13.1	13.2
4	16	9	1.00	1.00	21	25	4	5.1	1335	2355	15.1	11.6
5	29	28	3.00	2.67	14	14	1.4	1.4	710	710	27.6	40
6	23	^#^	1.33	^#^	17	^#^	2	^#^	1070	^#^	21.7	^#^
7	9	9	0.33	0.33	27	27	3.5	6	1000	2940	9.7	8.4
8	20	18	1.00	0.67	17	18	3.5	4.2	1640	1930	15	14.7
9	19	17	2.67	2.00	19	22	2.2	3.3	1100	1230	16.3	15
**Mean**	**21.1**	**17.8**	**1.8**	**1.5**	**17.3**	**19.8**	**2.7**	**3.8**	**1104**	**1669**	**21.7**	**19.4**
**SD**	**8.2**	**7.1**	**0.9**	**0.8**	**5.3**	**4.5**	**0.8**	**1.3**	**279.5**	**665.6**	**12.8**	**10.7**

Total scores of the Spastic Paraplegia Rating Scale (SPRS Sum), the Tinetti Balance and Gait Test (Tinetti Sum), and the Timed Up and Go Test (TUG), and the mean of the modified Ashworth scale (ASH Mean) before (pre) and after (post) treadmill training for each patient. The maximum speed (Max Vel., maximum velocity) and the maximum distance (Max Dist., maximum distance) were measured on the treadmill at the second (pre) and last (post) training session. Calculated are mean values and standard deviation (SD) for each test. Patient 6 could not take the clinical tests after treadmill training due to illness. Pat. No, patient number, ^#^no data available, m, meters, s, seconds, m/s meters per second.

### 3.2 Unperturbed stance

**FIGURE 2:**
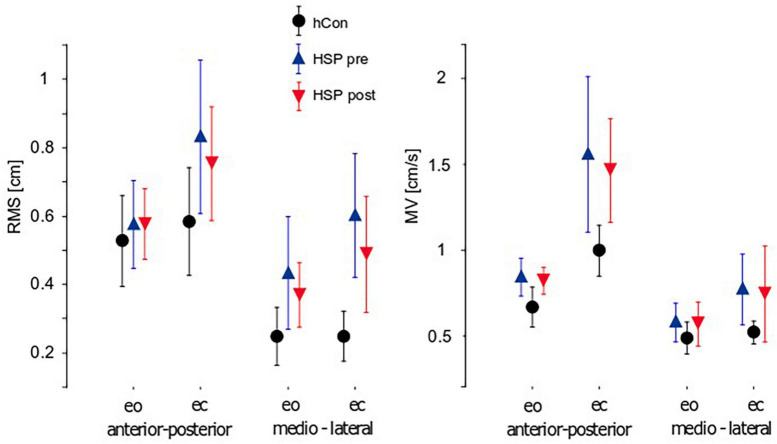
Parameters of unperturbed stance (mean ± 95% confidence interval). Root Mean Square (RMS), Mean Velocity (MV) and 50%-frequency of body sway (F50) of the control group (hCon) and the HSP group before (pre) and after (post) treadmill training with eyes closed (ec) and eyes open (eo) in anterior-posterior and medio-lateral sway direction. Hz, Hertz.

Results of unperturbed stance are presented for the center of pressure (COP; Figure 2).

Sway amplitude was quantified by calculating Root mean square (RMS). RMS of the HSP group was significantly larger compared to the control group (hCon; HSP 0.61 cm, hCon 0.40 cm, *F* = 20.7, *p* < 0.0001). Likewise, Mean Velocity (sway velocity, MV) was significantly larger in the HSP than in the control group (HSP 0.94 cm/s, hCon 0.67 cm/s, *F* = 16.5, *p* = 0.0001). Sway amplitude and velocity depended on visual condition with larger and faster sway upon eye closure (RMS: *F* = 7.0, *p* = 0.01; MV: *F* = 23.2, *p* < 0.0001). Group differences were significantly more pronounced during trials with eyes closed (RMS: *F* = 4.1, *p* = 0.048; MV: *F* = 4.2, *p* = 0.04). Sway amplitude and velocity were also more pronounced in anterior-posterior (a-p) than in medio-lateral (m-l) direction (RMS: *F* = 29.1, *p* < 0.0001; MV: *F* = 41.6, *p* < 0.0001). We observed no interaction between groups or sway directions.

Two weeks of daily treadmill training reduced the HSP group’s sway amplitudes significantly (RMS: pre 0.63 cm, post 0.55 cm, *F* = 8.3, *p* = 0.008). Patients still failed to achieve the values of healthy controls. In contrast, MV did not significantly change due to training (MV: pre 0.97 m/s, post 0.90 m/s, *F* = 3.1, *p* = 0.09). Treadmill training did not significantly affect visual conditions or directions of sway.

### 3.3 Externally perturbed stance

**FIGURE 3:**
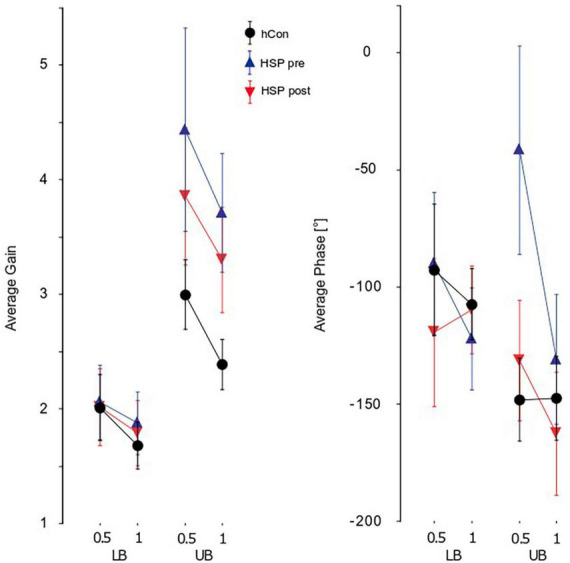
Parameters of perturbed stance. Group averages for GAIN, PHASE, and COHERENCE (mean ± 95% confidence interval) of the lower (LB) and upper (UB) body across both stimulus amplitudes (0.5°, 1°) and visual conditions (eyes open, eyes closed). The groups shown are the control group (hCon), and the HSP group before (pre) and after (post) treadmill training. F, frequency; Hz, Hertz.

Our HSP group’s GAIN values were significantly higher than the control group’s (HSP 3.0, hCon 2.4, *F* = 24.4, *p* < 0.0001). We detected a significant interaction between group and body segments, with more pronounced group differences at the upper body (*F* = 19.2, *p* < 0.0001; Figure 3). In contrast, stimulus amplitudes did not interact significantly with group. GAIN as function of stimulus frequency rose up to 0.2–0.4 Hz and fell rapidly at higher frequencies. At approximately 1 Hz, body angle and stimulus amplitude were equal (GAIN = 1). Treadmill training significantly reduced GAIN (pre 2.94, post 2.74, *F* = 4.3, *p* = 0.04) without reaching the values of control subjects. Treadmill training did not significantly influence GAIN with respect to body segments, frequencies or stimulus amplitudes (Figure 3).

We observed significant group differences between PHASE of the HSP and control group (HSP −96.1°, hCon −121.8°, *F* = 11.2, *p* < 0.001). As with GAIN, differences were more pronounced at the upper body with a significant interaction between groups and body segments (*F* = 22.4, *p* < 0.0001; Figure 3). Group also interacted significantly with stimulus amplitudes (*F* = 12.4, *p* < 0.001) which is due to the fact that the main group differences are observed in trials with 0.5° peak-to-peak amplitude. After 2 weeks of treadmill training, The HSP group’s PHASE values approached the control group’s values significantly (pre −91°, post −130°, *F* = 19.7, *p* < 0.0001). The training effect interacted significantly with the body segments (*F* = 10.1, *p* = 0.002), and stimulus amplitudes (*F* = 11.2, *p* = 0.0009; Figure 3). PHASE lag of the upper body increased from −78.8° to −130.8°, whereas the lower body’s PHASE lag increased from −103.3 to −114.6°.

The HSP group’s COHERENCE was significantly smaller than the control group’s (HSP 0.36, hCon 0.46, *F* = 109.0, *p* < 0.0001) reflecting the smaller signal-to-noise ratio due to the former’s larger unperturbed stance. Moreover, we found a significant interaction of group with body segment (*F* = 18.4, *p* < 0.0001) displaying larger COHERENCE differences between groups at the upper body. There was no interaction between groups and stimulus amplitudes. Treadmill training significantly increased the HSP group’s COHERENCE (pre 0.36, post 0.43, *F* = 119.0, *p* < 0.0001). It significantly affected the COHERENCE of hip and shoulder (body segments, *F* = 9.4, *p* = 0.002), but did not change the reaction to different stimulus amplitudes (*F* = 0.0, *p* = 1.0).

GAIN, PHASE, and COHERENCE were frequency-dependent in both the HSP and control group. However, we found no significant interaction between group and frequency.

### 3.4 Model-based analysis

The integral gain (Ki) of the HSP group was significantly smaller than the control group’s Ki (HSP 59.8, hCon 70.7, *F* = 8.03, *p* = 0.008). Time delay (Td) was significantly larger in the HSP group (0.21 s, hCon 0.18 s; *F* = 12.9, *p* = 0.001). The proportional (Kp) and derivative (Kd) gain, the proprioceptive weight (Wp) and passive gains Kpas and Bpas did not differ significantly between the HSP and control group (Kp: *F* = 0.9, *p* = 0.35; Kd: *F* = 3.0, *p* = 0.09; Wp: *F* = 0.002, *p* = 0.96, Kpas: *F* = 2.5, *p* = 0.13; Bpas: *F* = 2.6, *p* = 0.12).

**FIGURE 4:**
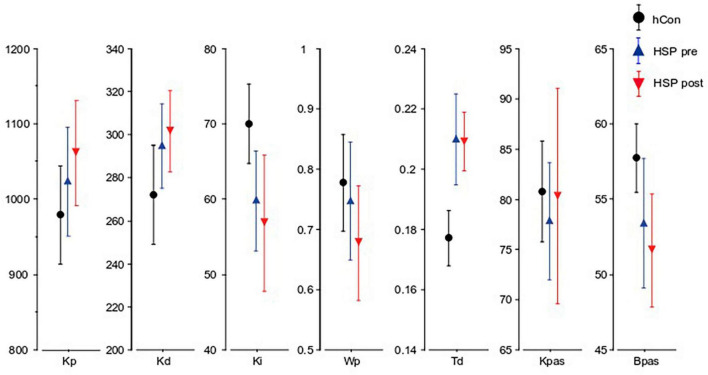
Parameters obtained from the model-based analysis. Kp (Nm ⋅ rad-1), Kd (Nm ⋅ s ⋅ rad-1), Ki (Nm ⋅ s-1 ⋅ rad-1), Wp, Td (seconds), Kpas, Bpas of the control group (hCon), and the HSP group before (pre) and after (post) treadmill training across all stimulus amplitudes with eyes closed.

Treadmill training seemed to have no significant effect on the model parameters (Ki: *F* = 0.05, *p* = 0.8; Kp: *F* = 0.4, *p* = 0.6; Kd: *F* = 0.001, *p* = 0.9; Wp: *F* = 1.0, *p* = 0.3; Td: *F* = 0.07, *p* = 0.8; Kpas: *F* = 0.3, *p* = 0.6; Bpas: *F* = 0.5, *p* = 0.5; Figure 4).

## 4 Discussion

In this study we compared the postural control mechanisms of patients with Hereditary Spastic Paraplegia (HSP) to a healthy control group taking a perturbation-based approach. Through a model-based analysis we aimed to identify functional correlates of postural impairments caused by pyramidal tract lesions. We also evaluated the effect of a “Structured Speed-Dependent Treadmill Training” (STT) protocol (on postural control).

Our ratings of SPRS and Ashworth scale indicate disease severities resembling those reported in studies by Schüle et al. (2006) and Martinuzzi et al. (2016) (SPRS 23.37 ± 10.7, and 20.0 ± 10.2, respectively; Ashworth Scale 2.04 ± 0.98). The values of the Timed Up and Go Test (TUG) in our study resemble the values of Heetla et al. (2015) who describe in an HSP patient’s case report that a continuous intrathecal Baclofen infusion reduced TUG values from 32 to 17 s. In our study, TUG values did not differ significantly, but maximum walking distance and speed improved significantly after treadmill training.

Prior to treadmill training, our HSP group’s body sway in terms of sway amplitude (RMS) and sway velocity (MV) was significantly greater than our control group’s. Our finding of increased RMS and MV stand in contrast to results of Nardone et al. (2001) who described a normal sway area in paraparetic patients. This discrepancy might be explained by differences in patient groups between the study of Nardone et al., and ours, as Nardone et al. (2001) included paraparetic patients with different diseases (HSP, idiopathic spastic paraparesis, sequela of resected meningioma). Large RMS and MV may be explained by an abnormally large sensorimotor feedback time delay and reduced long-term correction gain as explained in more detail below (see also Collins et al., 1995; Maurer and Peterka, 2005). Treadmill training induced less sway amplitude.

Hereditary Spastic Paraplegia patient’s postural adjustments, especially in the upper body after small platform tilts are more advanced in a timely manner than those of healthy control subjects. In addition, HSP patients react with larger body reactions and more random sway movements to platform tilts. Altogether, these findings might be interpreted as an affectation of intersegmental coordination.

It is well known that HSP mainly affects the largest and fastest conducting motor axons (e.g., McDermott et al., 2000; Martinuzzi et al., 2016). Accordingly, nearly all patients reveal increased motor conduction time (Jørgensen et al., 2005; Sartucci et al., 2007). With the help of our model-based analysis, we identified an increased time delay (Td) and reduced long-term error correction (Ki) in postural reactions of the center of mass in HSP patients. They may represent additional functional correlates of impairments due to pyramidal tract lesions. Treadmill training did not influence time delay, or long-term error correction of the center of mass. We suppose that the increased time delay is a functional correlate of the increased motor conduction time and thus may not be modifiable by any intervention. However, the changes of GAIN and PHASE values toward a normal range indicate that HSP patients learn to compensate for their functional deficits by adjusting parameters that are changeable. Moreover, the upper body’s phase lead was reduced.

Until now, the benefit of physical exercises such as treadmill training for patients suffering from HSP and other forms of pyramidal tract lesions seemed to be unclear. Adams and Hicks (2011) reported a beneficial effect of treadmill training on spasticity in patients with chronic spinal cord injury. In addition, robotic gait training significantly improved clinical assessments of gait and balance as well as quality of life in HSP patients, even after a 2-month follow-up period (Bertolucci et al., 2015). On the other hand, systematic reviews decry the lack of high-quality trials addressing the effect of physical exercise on spasticity in different neurological diseases (Ashworth et al., 2012; Khan et al., 2019). Mehrholz et al. (2017) found no evidence for a beneficial effect of treadmill training in stroke patients in their overall ability to walk. However, walking speed and endurance seemed to improve temporarily, especially in people able to walk (Mehrholz et al., 2017).

In conclusion, we have demonstrated that HSP clearly damages postural stability, specifically motor reactions that are more fragile and delayed. The long-term error correction of movements is also impaired. While treadmill training did not modify increased time delay and reduced long-term error correction gain of the center of mass, probably because of the disease’s unaltered anatomical correlate, i.e., pyramidal tract lesions, the specific profile of training benefits indicates that HSP patients’ postural control was ameliorated by compensatory mechanisms. We hold that our model-based analysis of sensorimotor behavior is capable of differentiating between functional correlates of a disease’s anatomical substrate and the parameter changes due to therapeutic interventions.

Study limitations are our small sample size and the lack of a control intervention. Hence, we cannot rule out that the beneficial effects on postural impairments are attributable to a regular and supervised exercise protocol rather than treadmill training *per se*. In addition, we did not assess the long-term effects of treadmill training. Further research with larger cohorts and follow-up examinations is needed.

## Data availability statement

The raw data supporting the conclusions of this article will be made available by the authors, without undue reservation.

## Ethics statement

The studies involving humans were approved by the Ethics Committee of the University of Freiburg (IRB # 256/01). The studies were conducted in accordance with the local legislation and institutional requirements. The participants provided their written informed consent to participate in this study.

## Author contributions

DD, BH, CW, and CM contributed to conception and design of the study. DD organized data acquisition and analysis. DD and CM wrote the first draft. IW and CM contributed to data analysis and further draft of the manuscript. All authors contributed to manuscript revision, read, and approved the submitted version.
